# Brexit anxiety: a case study in the medicalization of dissent

**DOI:** 10.1080/13698230.2018.1438334

**Published:** 2018-02-15

**Authors:** Dan Degerman

**Affiliations:** a Department of Politics, Philosophy and Religion, Lancaster University, Lancaster, UK

**Keywords:** Arendt, political emotions, political agency, EU referendum, Brexit

## Abstract

This paper illustrates how concepts of mental disorder have been deployed to medicalize negative emotions and, thereby, weaken the political agency of some individuals. First, I theorise the link between political agency and emotions, arguing that effective political action entails the transformation of emotions into public issues. Using the British referendum on membership in the EU as a case study, I then examine how medically loaded terms and rhetoric were used to describe suffering after the vote. Finally, I argue that this generated conditions that interrupted or even reversed the transformation of subjective experiences into politically meaningful issues.

## Introduction

Emotions occupy a central role both in political theory and practice. The so-called affective turn has been ongoing for more than a decade, engaging political theorists who seek to rehabilitate the role of emotions in politics (e.g. Ferry & Kingston, 2008; Goodwin, Jasper, & Poletta, 2001; Hoggett & Thompson, 2012). Whatever influence this academic debate has had on politics, it seems that emotions have, as one commentator put it, been ‘emancipated’ (Wouters, 2012), at least in a certain sense. Today, we expect our political leaders to display emotions publicly during certain events.^1^ And it is now a matter of course for politicians to appeal explicitly to the *hearts* as well as the minds of voters, in an apparent acknowledgment of the legitimate role that emotions play in politics (e.g. McIntosh, 2015). It is not surprising then that it has seemingly become more acceptable for average citizens to show emotions in particular social and political contexts as well.^2^

The emancipation of emotions has, arguably, increased the political agency of individuals whose attempts to participate in political discourse have long been dismissed or undermined on the basis that they are overly emotional and irrational, including women, black people, and members of the working class. The prevailing conditions seem to limit, though not prevent, attempts to delegitimize and disempower by appeal to the reason-emotion dichotomy. In this paper, I will theorize how another, increasingly influential dichotomy impacts political agency, namely, the dichotomy between healthy and disordered emotions.

Using examples from British newspaper articles, political commentary, and mental health blogs published in the period immediately before and after the referendum on British membership in the EU on June 23, 2016, I will illustrate how ideas of mental disorder have been used to medicalize the emotions of citizens and, thereby, weaken their political agency.

I begin by outlining the connection between political agency and emotions, drawing on Hannah Arendt’s ideas about political action. Emotions, I argue, must be transformed into a public issue to become a basis for effective political action. Within liberal democracies, the resources and opportunities required for such transformations are widespread, if unequally distributed and accessible. I then proceed to examine the treatment of negative emotions in the post-referendum discourse. In the aftermath of the vote, there were reports that people were supposedly flooding psychiatric clinics to receive care for their Brexit-related emotional suffering. Mental health experts and journalists dubbed this suffering ‘Brexit anxiety’. They warned that the anger, fear, and sadness that many people felt – especially those who had voted to remain – were incipient symptoms of mental disorder. To avoid full-blown disorder, people had to manage these emotions carefully. An effective means of management, according to numerous therapists and employers, was for people to recognize their powerlessness in the face of political events, and focus on their private lives. Some pundits also seized on the idea of Brexit anxiety, but for a different purpose, namely, to ridicule, shame, and delegitimize the actions of those who continue to oppose Brexit. In conclusion, I argue that the case of Brexit demonstrates how concepts of mental disorder in political discourse may interrupt or even reverse the transformation of emotions into public issues.

## Arendt, political agency, and emotion

Political agency in this paper will refer to the individual’s capacity to act in concert with other people to shape or respond to a public issue. This definition draws on the thought of Hannah Arendt, according to whom power arises from action in concert. In this view, the power generated in collective action can be used to establish and preserve political institutions and laws, but also to challenge, reform, and destroy them (Arendt, 1961, p. 153; 1972, p. 56). The term public issue is owed to Mills (1959, p. 187), and refers to a problem that an individual shares with others and which can only be solved through structural change on a group, community, or societal level.^3^ It also fits well with and clarifies Arendt’s central claim that political action always concerns ‘men in the plural’, never ‘man in the singular’ (Arendt, [1958] 1998, p. 4).^4^

As I indicated above, I am interested in how the medicalization of negative emotions impacts our capacity to take political action.^5^ Since this presumes that there is a connection between emotions and political agency, I should clarify what this connection is. However, Arendt is not exactly famous for her odes to emotional politics. Quite the contrary. Among scholars of political emotion Arendt is perhaps best known for her critique of emotion. They have taken her to claim that emotions *ought* to be kept in the private sphere, away from the public realm in which politics occurs. I have argued against this interpretation at length elsewhere (Degerman, 2016). Basically, I claim that Arendt’s worries about emotions in politics are rooted in an idiosyncratic understanding of emotion as a combination of wordless, visceral reactions and radically subjective experiences. These experiences are, according to Arendt, inherently hidden within the heart’s darkness, and attempts to externalize and use them in the public sphere have in the past engendered a destructive fixation with authenticity.

Many of us today have a more expansive understanding of what an emotion is than did Arendt. While we might agree with her that subjective feeling is an essential part of an emotion, contemporary philosophers have convincingly argued that other components – such as judgments, words, and deliberate actions – are also part of what constitutes an emotion (Lupton, 1998; Reddy, 2001; Solomon, 2004). Why then am I using Arendt to outline the connection between political agency and emotion?

Despite its limitations, Arendt’s thinking contains an insight that is frequently obscured in contemporary debates on political emotions and action. Namely, emotions and other subjective experiences are *not* inherently political. They must be turned into something political, that is, into something that we can share and act on with others. As Arendt puts it in *The Human Condition*:

… the greatest forces of intimate life – the passions of the heart, the thoughts of the mind, the delights of the senses – lead an uncertain shadowy kind of existence unless and until they are transformed, deprivatized and deindividualized, as it were, into a shape to fit them for public appearance. (Arendt, [1958] 1998, p. 50)

She suggests that such transformations are facilitated by a common world – a shared world of things, speech, and relationships (Arendt, [1958] 1998, pp. 50, 168). The common world is a central condition of political action in Arendt’s work, much of which is dedicated to exploring the relationship between the two. However, she does not pay much attention, either in *The Human Condition* or in later works, to how the common world enables the transformation of subjective experiences into a shape ‘fit for public appearance’. Instead, she is preoccupied primarily with defining the proper limits of the public contra the private sphere, almost as though their mere existence guaranteed the possibility of political action. This has led some critics to assert that Arendt simply presumes the existence of ready-made political agents who are ready to act as long as there is a public sphere. McNay (2014), for example, accuses Arendt of overlooking many of the obstacles to political action that exist within the public sphere, and their capacity to affect people differently depending on gender, race, class, and capacity. Individuals and groups who find themselves surrounded by such obstacles often end up internalizing injustice as negative emotions directed at their individual lives, and end up blaming themselves for their misery. McNay rightly observes that by ‘being incorporated in this manner, the social origins of suffering are obscured and are experienced instead as the fault of the individual’ (p. 115).

This criticism is partly justified. But some of Arendt’s earlier work suggests she understood how disempowering the inability to transform subjective experiences into a shape fit for ‘public appearance’ could be. One of the books that supports this claim is Arendt’s ([1957] 1997) biography of Rahel Varnhagen – a Jewish woman, who at the turn of the eighteenth century hosted one of Berlin’s most popular salons.^6^ With the rise of Napoleon and antisemitism, Varnhagen fell out of favor with high society, and she spent most of her remaining life trying to regain her standing. Arendt’s biography focuses on this latter part of Varnhagen’s life. The work can be read as the tragic story of a Jewess who in her single-minded and self-centered pursuit to improve her status in society failed to understand the connection between her personal suffering and the political problems of Jews in general.

In *The Origins of Totalitarianism*, Arendt identifies the same tendency towards individualization more broadly among the pre-war European Jews. There she links it more explicitly to the structural problems faced by Jews who sought assimilation. They were required on the one hand to distinguish themselves from the average Jews and their cultural and racial taint. On the other, they also had to distinguish themselves from non-Jews to prove that they had something to contribute to the society that admitted them and were thus worthy of its protection (Arendt, 1958, pp. 65, 66). The very possibility of assimilation, in effect, entailed a recognition of individual responsibility and a denial of the political meaning of the suffering of Jews. Reflecting on this, Arendt says: ‘The adage, “a man in the street and a Jew at home,” was bitterly realized: political problems were distorted to the point of pure perversion when Jews tried to solve them by means of inner experience and private emotions’ (p. 67).^7^ The grounds for solidarity and effective political action among Jews were thus severely undermined (pp. 117, 118), if not completely destroyed (p. 120). They did not lose the capacity to speak about their problems as such of course; Arendt observes that during the many years of persecution, the Jews developed a rich cultural life which helped them to survive (1968, pp. 12, 13). What they lost or lacked were the concepts and resources to transform their problems into a political shape, that is, into public issues.

These examples illustrate that without the capacity to relate our subjective experiences to worldly conditions – matters that we share with others – political action becomes impossible because it appears that there is nothing that we can act on together.

## Transforming negative emotions into public issues

The notion that political action entails a transformation of emotion resonates with how other political thinkers and activists have described the experience of acting politically. Several have used terms closely related to transformation, such as ‘transition’, ‘transmutation’, and ‘channeling’. These are often applied to anger, although I think the concept of transformation is applicable to at least all negative emotions. Notably, Mahatma Gandhi and Martin Luther King both spoke in these terms. Gandhi (1999, p. 252) once said: ‘I have learnt through bitter experience the one supreme lesson to conserve my anger and as heat conserved is transmuted into energy, even so our anger controlled can be transmuted into a power which can move the world’. In an apparently matching spirit, Martin Luther King (1986, p. 49) relayed his experiences of dealing with negative emotions: ‘As my sufferings mounted I soon realized that there were two ways that I could respond to my situation: either to react with bitterness or seek to transform the suffering into a creative force’.

Leaders within the second wave feminist movement also spoke in terms suggesting that women’s negative emotions had to be deliberately transformed into something political. The civil rights activist and black feminist writer Audre Lorde spoke eloquently – and passionately – about this need: ‘Every woman has a well-stocked arsenal of anger *potentially useful* against those oppressions, personal and institutional, which brought that anger into being. Focused with precision it can become a powerful source of energy serving progress and change’ (1984, p. 127; emphasis mine). However, Lorde observed: ‘Most women have not developed the tools for facing anger constructively’ (p. 130). Consciousness-raising sessions were central to developing these tools (at least among white women), that is, concepts and relationships that allowed women not just to speak, but to be heard and seen where they had previously been silent and invisible. As another activist-scholar put it, these sessions enabled women ‘to translate their individual feelings of “unfreedom” into a collective consciousness’ (Freeman, 1973, p. 800). McNay (2014, p. 115) similarly describes how consciousness-raising groups translated ‘personal experiences of suffering into an impersonal analysis of [women’s] subordination’. The consciousness-raising groups provided space, affiliations, and concepts – empowering factors – which permitted women to transform subjective experiences into a public issue. Thereby, women’s individual experiences were imbued with shared meaning, and their concerted action was directed toward specific problems, such as workplace discrimination, sexism, etc.

Nussbaum (2016), in her recent book *Anger and Forgiveness*, describes a comparable, but narrower, process of moving from anger to political action, which she calls transition. It is worth distinguishing briefly between her idea of emotional transition and my idea of emotional transformation. For whereas Nussbaum’s transition implies that the original emotion has been left behind, my Arendtian concept of transformation presupposes little about the mental life of the actor, except that the actor herself believes that she has experienced an emotion and that her understanding of its basis has shifted into shared terms. Transformation in my rendering, hence, permits the emotion to persist and continue to fuel the action. By contrast, Nussbaum demands that the anger be purged and replaced by thoughts about the common good, a demanding requirement with few evident benefits.^8^

The kinds of empowering factors that were present in the feminist consciousness-raising groups, which allowed individuals to transform emotions into shared concerns, are not unique to social movements. Such resources are relatively common within liberal democracies. Even the more formalized aspects of democracy – such as general elections and referenda – provide factors that facilitate transformation of emotions, that is, affiliations, spaces, things, concepts, and so on, through which (some) citizens can transform their emotions into political views and action in concert.

This feature of political space and discourse became patent in the period leading up to the British referendum on EU membership last year, an issue which became commonly known as Brexit. Now, according to cognitive scientists and political psychologists, emotions play a central part in all human decision-making, not least when it comes to deciding whom or what to vote for. But in the Brexit debate, the role of emotions was more explicit. Campaigners on both sides seemed to talk about emotions incessantly (e.g. Brolin, 2016; Elliott, 2016; Heffer, 2016; Johnson, 2016; Smyth, 2016). Whereas their opponents were ‘appealing to the baser instincts of voters’, they were ‘making the passionate case’ for their side (Heffer, 2016). Either way, a prominent school of thought held that ‘Brexit [was] not about facts, it [was] about feelings and emotions’ (Schama, 2016).^9^

The Brexit debate generated public discourses and spaces in which (many) people (but not all) could transform their anger, fear, and sadness into political views: for or against immigration, for or against supranational laws, for or against Brexit, and so on. Although the room for nuance or compromise within these spaces was generally narrow, perhaps even non-existent, many people (especially on the winning side) clearly felt empowered by the process and the outcome – to many on the Leave side it was after all a question of ‘taking back control’.

These initial remarks – which hopefully are not too controversial – are intended as a clarification of how I understand common political phenomena. As mentioned at the outset, I am interested primarily in what happens to our capacity for political agency when negative emotions are medicalized. We shall see that the aftermath of the Brexit referendum provides an interesting case study through which to explore this question. I will argue that the medicalization of emotional re-actions to the referendum undermined the actual and potential connections between emotions and political issues associated with Brexit, in some cases with the apparent intention of silencing dissent. That is, I am theorizing that medicalization in the post-referendum discourse had both immediate and long-term effects. The immediate effect was to undermine the political agency of some people with respect to Brexit and related issues. The more long-term effect was to reinforce politically disempowering factors that frustrate and sometimes even thwart the transformation of powerful negative emotions into public issues.

## A brief note on method

The strong emotional overtones to the Brexit debate are what drew me to examine it in the first place, as part of a broader project exploring how the medicalization of negative emotions impacts political agency. I had reasons to expect a rich object of study, but could of course not be certain of what I would find and its relevance to the project. The process began with a search for the term ‘Brexit’ together with some common mental disorder-words like ‘anxiety’ and ‘depression’ in the ProQuest newspaper database. One of the first pieces that appeared in the search was a news article in *The Evening Standard* headlined ‘“Brexit anxiety” brings queue of patients for psychiatrists’, published shortly after the referendum, along with a number of the newspaper articles that I discuss herein. I then attempted to follow the trail as I saw it: The news articles cited some mental health blogs to support their claims – what did these actually say, and were other mental health professionals writing similar things? Who else used the term ‘Brexit anxiety’, or other mental health terms in relation to Brexit, and for what purpose? Following the trail that the initial news articles set me upon, and answering these questions, led me beyond the initial resource to other publicly available materials, such as opinion pieces, blog posts, YouTube clips, and organizational documents. The result is the case study I present below, which highlights one strand of medicalizing discourse. This is not an attempt at quantitative analysis. Hence, my approach does not allow for any precise statement as to how important or prevalent the medicalizing discourse was overall in the EU referendum. It does, however, permit me to illustrate how this and similar strands of medicalizing discourse can impact political agency.

## ‘Doctor, I think everyone has Brexit anxiety’

The day after the EU referendum, on Friday, June 24, 2016, the votes had been counted and a winning side declared; 51.9% of voters opted for the UK to leave the EU. London, Scotland, and Northern Ireland had overwhelmingly voted to remain, whereas much of non-metropolitan England and Wales voted to leave. Prime Minister David Cameron resigned in response to the defeat of the side he had led. In the days and weeks that followed, massive protests materialized on the streets of major cities in which people expressed sadness, fear, and, most prevalently, anger about the result and its potential consequences. These were people ready to act in the face of what many of them saw as a threat to the UK, Europe, and the world.

Though the referendum was over, critical political questions remained to be addressed. The most general and important of these was: What did Brexit actually mean? Despite this and other pressing issues, the new prime minister, Theresa May, her government, and its supporters appeared intent on challenging the legitimacy of any further debate or public involvement on Brexit. Differently put, they attempted to depoliticize Brexit, sometimes through methods both banal and absurd. Theresa May, for example, adopted the empty mantra: ‘Brexit means Brexit’. The intense and public emotions that had been generated in the preceding months potentially imperiled these efforts. However, as we shall see, this problem began to resolve itself as people began to claim that these emotions were perhaps not political after all.

Immediately after the EU referendum, the number of people in therapy was supposedly surging, according to news reports. Some individuals had apparently been so upset by the referendum result that they required professional mental health care. A headline in *The Sun* claimed that psychiatrists had seen an ‘alarming rise in [the] number of patients seeking help for “Brexit anxiety”’ (Lockett, 2016). Another tabloid suggested that ‘Brexit anxiety’ had brought patients queuing for mental health care (Prynn, 2016).

Such assertions were not confined to the tabloids, although their claims were the most hyperbolic. *The Guardian*, *The Financial Times* and several other news outlets also suggested that the referendum had increased the demand for mental health care (e.g. Court, 2016; Jacobs, 2016; Orbach, 2016; Watts, 2016). An opinion piece in *The Daily Telegraph* even implied that the increased pressure was pushing psychiatrists to the point of breaking (Fitzpatrick, 2016).

What evidence did they have to support these claims? Not much. Among the articles I have surveyed, only one cited evidence of an increase in patients. The article in question quoted a staff member at a private mental health institution in London, who claimed that a recent rise in patients was directly attributable to the referendum outcome (Moore-Bridger, 2016). Most other papers appear to have relied primarily on blog posts and opinion pieces written by mental health professionals.^10^ Among the most notable expert writers on this matter was the renowned feminist author and psychotherapist Susie Orbach. In a piece for *The Guardian*, headlined with her name and the phrase ‘in therapy, everyone wants to talk about Brexit’, she described the volatile emotions that her clients had expressed about the outcome of the referendum, such as anger, fear, and – especially – anxiety (Orbach, 2016). Other mental health professionals related comparable experiences on their blogs and professional websites. But none of them mentioned anything to support the claim that the number of patients was surging. What some did report was that most of their existing patients wanted to talk about Brexit.

While the queues of psychiatric patients might have been a figment of the journalistic imagination, the idea of Brexit anxiety was not, or at least not entirely. The term seems to have first appeared in 2015, when it was used to describe the tendency of investors to withhold or withdraw investments from the UK due to the economic uncertainties that Brexit meant for businesses and the national economy. In this context, the word ‘anxiety’ evidently did not refer to any subjective experience we might have or to any mental disorder we might suffer from; rather, it served as a shorthand for a particular economic indicator, and this continued to be its primary usage in the months after the referendum.^11^ Arguably, however, this usage did not completely sever the word from its emotional and psychiatric connotations, which may be significant. Describing the economy using emotion terms like anxiety and fear can invite us to think of it as irrational, as Berezin (2009, pp. 335–337) has observed, or perhaps even as insane (Lewis, 2008; cited in Berezin, 2009).^12^ When applied to the economy and other institutions that are usually surrounded by a façade of rationality, such terms may be politicizing and empowering. But these means are a double-edged sword. Much like anxiety can open opportunities to intervene on institutions, it can open opportunities to intervene on individuals. And the move from thinking about institutions as irrational to thinking about individuals as irrational is an easy one; after all, if an institution is irrational does that not suggest that the individuals working within it are acting irrationally? Hence, the deployment of the term Brexit anxiety in the economic context seems in a sense to have paved the way for the explicitly medicalized meanings and force it assumed in the hands of mental health professionals.

## The therapist’s guide to getting over Brexit

Throngs of experts jumped on the chance to offer their analyses of and advice on the mental health of the British population after the referendum (e.g. Burgess, 2016; Cullen, 2016; Kurz, 2016; Private Psychiatry, 2016; Rowland, 2016; Sanderson, 2016; Sieger, 2016; WPF Therapy, 2016). Of special interest were those who had voted to remain and found themselves on the losing side of one of the most emotional and vicious political campaigns in the history of British democracy, and who were consequently the most vulnerable to the so-called Brexit anxiety.

The apparent meaning of this term varied somewhat. Some experts deployed it to describe the worry that people might have about the consequences of the referendum. Others used it loosely to encompass a range of unpleasant emotions that people might feel due to the referendum, including anger, fear, and sadness. Common among them was the idea that Brexit anxiety was an incipient form of mental disorder that needed to be managed to avoid serious health consequences. The emotional anxiety and depression people felt in the wake of Brexit were ‘symptoms’ that should not be ignored; as one for-profit psychiatry clinic urged on its website:

They are the biggest causes of mental health problems and one of the main reasons patients come to our practice – they can even cause further problems with physical health if not recognised and treated properly. (Private Psychiatry, 2016)

Mental health experts were of course happy to offer their advice on how to manage these dangerous emotions – and those who worked at places like Private Psychiatry had a clear financial incentive for doing so.

Interesting from a political standpoint is that a central element in many Brexit anxiety management plans was the notion that the individual had to acknowledge her lack of control in the face of Brexit, and to focus on ‘what really matters’ – like family and friends, as well as personal and professional success.^13^

Among the most colorful examples of this was a YouTube video in which a therapist showcased his avowed ability to help people to ‘get over Brexit’ using neuroscience-based hypnotherapy. The video shows a client, a middle-aged man, who begins by describing his anger over the referendum outcome. The man explains that he is distraught about the outcome of the referendum and how the Brexit debate has divided the country. Halfway through the video, we are to understand that treatment has transpired and the therapist prompts the man to explain how the treatment has helped him. The man responds:

It’s made me realise that I can’t do too much about it and it is really what it is. And we need to hope that things can become better. And for me to just accept it and let it go. (Cullen, 2016)

What is remarkable in this example is not the treatment, which is obviously outlandish. Most serious therapists, no matter how well-versed in neuroscience, would avoid claims that results like this were possible in a single session. What is striking rather is the patient’s response, which is portrayed as the ideal treatment outcome: the patient has realized that he can do nothing about the state of the country; all he can do is to accept it and hope it gets better. Apparently, the cure for his troublesome feelings about the state of the public realm is the insight that he is politically impotent.

The effect of this and other Brexit anxiety management plans I have come across seems to be to reverse the transformation from subjective experience to public issue. They explicitly guide people to sever the connection they have made between their emotions and politics, under the premise that failure to do so may lead to serious disease. If we believed that our emotions were tied to Brexit and its consequences, we were mistaken. What we must understand, these experts urge us, is that politics is not the cause of our suffering: we are.

## Brexit anxiety off the couch

‘But what’s the problem?’ you might ask. ‘Few people read mental health blogs. Those who do read them probably have a history of mental health problems, and might be especially vulnerable to the emotional fallout of political events’. But as I have already noted, news media also picked up the idea of Brexit anxiety and warnings of other dangerous emotional (over-)reactions to the referendum from these experts, disseminating them far beyond the regular readership of therapy blogs. In some news articles, Brexit anxiety even evolved from an incipient if dangerous symptom to a full-blown disorder. For example, at the bottom of *The Sun* report on the ‘alarming’ spread of Brexit anxiety, an information box titled ‘The Official NHS Doctors [sic] Guide to Anxiety’ provided a list of the symptoms and neurological causes of anxiety disorder (Lockett, 2016). In this way, *The Sun* and other publications also propagated the view that people had to manage their Brexit-related emotions carefully to avoid mental disorder.

Neither did these ideas merely bounce around in a media echo chamber. After the referendum, several organizations started offering special mental health support for staff who needed help to cope with Brexit. Some universities arranged group-counseling sessions for this purpose. For example, staff at the University of Nottingham who had ‘concerns about the potential changes following the Brexit decision and wish[ed] to enhance the ways in which they manage their own well-being’ could attend a half day workshop where they could learn ways ‘to navigate the uncertainties of political changes’ and ‘how to feel more in control when face[d] with uncertainty’ (The University of Nottingham, 2016). In an analogous effort, the University of Leeds published on its website a guide for dealing with the emotional reactions to Brexit, such as grief, anger, and sadness. The ‘tools and strategies’ contained in the guide were, not surprisingly, almost identical to those we saw in the mental health blogs. They included: appreciating the things one has, limiting exposure to news, avoiding agitating situations, and paying careful attention to the signals and needs of one’s body (Staff Counselling & Psychological Support Service, 2016). Given this, it seems likely that the consequences of the medicalization of Brexit anxiety and other emotions extend beyond those with a history of mental disorder.

It is also important to note that one need not buy into these kinds of medicalized conceptions of emotions to be affected by them politically. Among some publications, the phenomenon of Brexit anxiety, the provision of therapy for those supposedly suffering from it, and the general use of a mental health vocabulary to describe the distress among Remain voters, became a means to delegitimize political opponents. After the referendum, the *Daily Express* and *Breitbart*, for instance, both highlighted conversations in online forums between young people who had voted to remain. Forum posts showed individuals describing themselves as ‘grieving’, ‘sick’, and feeling ‘genuinely depressed’, leading the *Daily Express* (2016) to assert – without evidence – that thousands of ‘whinging students are complaining they’re suffering from depression’. Drawing on the same forum discussion, a *Breitbart* article proclaimed that ‘students “depressed” and “traumatised” by Brexit say they will fail exams’. The same article also accused students of holding ‘firmly anti-democratic views’ (Deacon, 2016).

Some politicians and pundits made similar claims. An NHS trust in southeast England was among the first workplaces to announce that it would provide free mental health support for staff emotionally affected by the outcome. Reacting to the announcement, the UKIP MEP Jane Collins called the initiative ‘an insult to democracy and an insult to people who expect their NHS to deliver healthcare for sick people not those having referendum-related tantrums’ (Stevens, 2016). Whereas mental health bloggers generally sought to characterize the emotional fallout on Brexit as shared by both sides, Collins had no time for such pretenses. In her view, it seems, only a loser would have reason to feel upset. Collins also stated plainly what Breitbart had implied: people who respond to Brexit with emotional tantrums of dislike are undermining British democracy and its core institutions. The previously mentioned initiatives at the universities of Leeds and Nottingham provoked comparable reactions when they came to light. A contributor on *The Conservative Woman* blog remarked:

Democracy has proven too hard to stomach for University of Nottingham academics[...] Sulking snowflakes will enhance their ‘skills for resilience in response to the Brexit decision’ by having half a day off work to sit in a room and moan about the grubby lower classes who upset them in June. (Anon, 2016)^14^

It is not that the harsh rhetoric and *ad hominem* attacks on people who voted to remain are surprising. Pundits of every stripe have been known to attack their opponents’ identities and right to a fair hearing instead of their arguments. The reason the attacks examined above are noteworthy is because they show how pundits have used the medicalization of post-Brexit emotions as a political weapon, deploying terms and ideas akin to those of mental health experts. Both the pundits and the experts claimed, in effect, that the strong emotions some people have experienced in the wake of the referendum are irrational or damaging feelings resulting from individual flaws rather than legitimate political concerns. And although therapists and pundits disagreed on the specific methods by which these individuals should overcome their flaws and what they should be called, their message is essentially the same: ‘Get over it. Move on’.

## Conclusion

Earlier I proposed that democratic elections tend to generate resources through which individuals can transform emotions into public issues. This capacity to transform emotions, especially negative emotions, is central to political agency. The reason we decide to act politically is usually because we perceive some wrong in the world, something that evokes emotions such as sadness, fear, or anger. If these emotions were not powerful, we would not act on them. Worryingly, it is precisely these kinds of powerful negative emotions that tend to be subject to medicalization.

The sort of discourse explored above, with the concept of Brexit anxiety at its center, interrupts or even reverses the transformation of emotions into public issues by calling into question the political meanings of our own and others’ emotions. Of course, doubts will always arise when we try to transform subjective experiences into shared terms – when we, so to speak, try to make the invisible appear. By exploring these experiences with others in relation to the world we have in common, to our political world, we can allay these doubts; together, we can identify the worldly causes of subjective suffering and act against them.

By transforming emotions into psychiatric symptoms or diagnoses, medicalization also allays doubts about subjective experience – probably much more effectively than the collective actions of regular citizens can. The authority of psychiatric explanation is so strong that it tends to exclude any competing explanations, including political kinds. Indeed, my ongoing research indicates that points of view challenging the validity of particular psychiatric diagnoses or treatments are often labeled anti-psychiatry and, hence, anti-science – which is basically just a different term for irrational (Degerman, 2017b). Moreover, medicine and psychiatry generally asks us to look for problems in ourselves – or, preferably, to let experts do it – and to change ourselves through medications or therapy to deal with these problems. As we have seen, such treatment regimens may be directly anti-political, asking patients to recognize their political impotence and to focus on their private sphere. That is to say, rather than guide us toward public issues, they drive us to conceive of our problems as – to borrow another term from Mills (1959, p. 8) – private troubles, which matter only to us and our doctor.

The Brexit anxiety discourse may not undermine the self-perceived right of campaigning political activists to participate in the political debate on Brexit, at least not directly. Few activists out on the street would buy into the view that they are suffering from mental problems as opposed to experiencing politically relevant and justified emotions. After all, in the immediate aftermath of the referendum, we saw large-scale protests, and since then groups have engaged in concerted actions on a smaller scale. Yet medicalized narratives can still sway those who occupy the middle ground, and exacerbate the rift between protestors and ‘regular’ people who do want to ‘move on’ – or have heard that they should be moving on – with their lives. A companion mantra to ‘Brexit means Brexit’ has been ‘the British people want us to get on with Brexit’. To these people, it may well seem that only mentally troubled individuals become so upset about politics that they take action, on the streets or elsewhere. Normal, healthy citizens do not cause a stir. They leave politics to the politicians. They keep calm and carry on.

## Disclosure statement

No potential conflict of interest was reported by the author.

## Funding

This work was supported by Wellcome Trust [108539/Z/15/Z].

## Notes on contributor

***Dan Degerman*** is a PhD candidate in philosophy at Lancaster University. His current research investigates how the medicalization of negative emotions impacts political agency.

## Notes

1. The importance of emotional displays in politics is clearest in cases of failure. For example, John Kerry’s loss to George Bush in the 2004 US presidential election has been explained in terms of the former’s inability to convey emotional authenticity to voters (Berlant, 2005). Similarly, in UK snap general election of 2017, Theresa May was derided for her unemotional style, earning the moniker ‘Maybot’ in the media (e.g. Asthana & Mason, 2017). But there are also some notable cases of successful emotiveness in politics, such as Tony Blair’s emotion-laden speech at Princess Diana’s funeral in 1997, which, as one commentator put it, ‘set the tone’ and ‘laid the foundations for much more than the dignified burial of Diana’ (Braidotti, 1997; see also Dixon, 2015, p. 302).

2. In his fascinating history of weeping in the UK, Thomas Dixon suggests that the death of Princess Diana was ‘the symbol and the harbinger of a new emotional era’ characterized by public displays of feeling (2015, p. 300).

3. A useful definition of political agency should exclude some types of behavior even if they are concerted in some sense, such as playing team sports, walking to work, and so on. Some theorists may want to categorize these behaviors as incipiently political because they reproduce power structures. Walking to work, for example, can be said to sustain the political order since it involves people conforming to and performing norms in view of others. But while these and some other types of behavior might have political ramifications, there are good reasons to define political action narrowly enough to exclude them. One of these is that it seems desirable to have a definition of political agency that permits individuals to, in Arendt’s (1972, p. 206) words, ‘know when power is lying in the street and when they can pick it up’. An excessively broad conception of political agency, wherein everything is political – as postmodernist theorists often seems to suggest – can have the opposite effect, leaving individuals unable to perceive any opportunity for meaningful political action. The definition I have proposed therefore deliberately constrains the scope of political agency to apply to the capacity to act together with others specifically with regard to public issues. This implies that to act politically, an individual needs not only to perceive some matter as political, but also to have access to other people who perceive this matter as political as well and be willing to act with this individual.

4. Pitkin (2006) also makes the connection between Arendt’s idea of political action and Mills’ concepts of public issues and private troubles.

5. Medicalization, according to Conrad’s (1992, p. 209) widely used definition, is ‘a process by which nonmedical problems become defined and treated as medical problems, usually in terms of illness or disorders’. It can sometimes be difficult to say when a problem or experience has been ‘medicalized’. Medicalization is a dynamic process; it does not always occur top-down, although critics often try to depict it that way. As Conrad (2005) has emphasized in recent work, there are several engines of medicalization, including medical experts, but also grassroots groups and public figures. These engines sometimes work in different directions – one pushing for medicalization while another is resisting it. Neither the claims of some experts nor the assertions of a few media pundits show that a problem or emotion has been fully medicalized, and that is not my claim in this paper. Rather, my claim is that some negative emotions were subject to a multipronged discourse of medicalization, driven by several engines – mental health experts, journalists, pundits, politicians, and workplaces.

6.
*Rahel Varnhagen: The Life of a Jewess* was first published in English in 1957, but Arendt had completed most of the manuscript by 1938 (Young-Bruehl, 1982, p. 91).

7. See note 6 above.

8. For a longer consideration of Nussbaum’s argument about transition and anger, see my review of *Anger and Forgiveness* in *Contemporary Political Theory* (Degerman, 2017a).

9. See also Reade (2016) and Mail on Sunday (2016).

10. Several of the mental health blog posts that I cite here were also cited as the main sources for claims about surging patient numbers in the newspapers.

11. A search in the ProQuest newspaper database, using the search string ‘“Brexit Anxiety” AND (psychological OR mental OR psychiatric OR therapy)’, indicates that Brexit anxiety was not used to describe individual mental health before 11 July 2016.

12. Interestingly, Berezin (2009) also highlights that the *Chicago Board Options Exchange Volatility Index* – which tracks volatility in the American stock market – is colloquially known as the ‘fear index’ among people in the finance industry and beyond.

13. For example, Rowland (2016), Sanderson (2016), LeBon (2016), and Cullen (2016) all offer variations on this advice.

14. For another similar example see Mikelionis (2016).
